# Impact of Cocoa Products Intake on Plasma and Urine Metabolites: A Review of Targeted and Non-Targeted Studies in Humans

**DOI:** 10.3390/nu11051163

**Published:** 2019-05-24

**Authors:** Ana Lucía Mayorga-Gross, Patricia Esquivel

**Affiliations:** 1 Centro Nacional de Ciencia y Tecnología de Alimentos, Universidad de Costa Rica, San Pedro 11501-2060, Costa Rica; 2 Escuela de Tecnología de Alimentos, Universidad de Costa Rica, San Pedro 11501-2060, Costa Rica; patricia.esquivel@ucr.ac.cr

**Keywords:** cocoa, chocolate, metabolites, biomarkers, metabolomics, urine, plasma, procyanidins, methylxanthines, polyphenols

## Abstract

Cocoa is continuously drawing attention due to growing scientific evidence suggesting its effects on health. Flavanols and methylxanthines are some of the most important bioactive compounds present in cocoa. Other important bioactives, such as phenolic acids and lactones, are derived from microbial metabolism. The identification of the metabolites produced after cocoa intake is a first step to understand the overall effect on human health. In general, after cocoa intake, methylxanthines show high absorption and elimination efficiencies. Catechins are transformed mainly into sulfate and glucuronide conjugates. Metabolism of procyanidins is highly influenced by the polymerization degree, which hinders their absorption. The polymerization degree over three units leads to biotransformation by the colonic microbiota, resulting in valerolactones and phenolic acids, with higher excretion times. Long term intervention studies, as well as untargeted metabolomic approaches, are scarce. Contradictory results have been reported concerning matrix effects and health impact, and there are still scientific gaps that have to be addresed to understand the influence of cocoa intake on health. This review addresses different cocoa clinical studies, summarizes the different methodologies employed as well as the metabolites that have been identified in plasma and urine after cocoa intake.

## 1. Introduction

### 1.1. Theobroma cacao L. composition

Cocoa (*Theobroma cacao* L.) is a tree from the Malvaceae family. Its seeds are covered by a sweet and sour mucilage which contains approximately 11% of sugars, mainly sucrose, and an acidic environment with a pH of about 3.5–3.8. Citric acid is the main organic acid present in the pulp. Others such as oxalic, phosphoric, malic and tartaric acid are also present [1,2,3,4,5,6].

Fat is the main component of cocoa beans: it accounts to almost 50% of the cotyledon dry weight, where 98% corresponds to neutral lipids, and 2% to polar lipids, mainly phospholipids and glycolipids. It has been reported that the major cocoa fatty acids are palmitic, stearic and oleic [7,8].

Proteins represent 17–20% of the dried bean [9,10]. Vicilin-like globulins and albumins are the two most abundant proteins and are key factors in quality development during fermentation. Concerning the amino acid profile, glutamic acid and aspartic acid showed the highest contents while cysteine showed the lowest ones, in both fermented and non-fermented cocoa derived products [11].

From a functional point of view, cocoa has been considered an important source of different bioactive compounds. The more important groups of compounds present in cacao are flavonoids, mainly flavan-3-ols, phenolic acids, methylxanthines, peptides, *N*-phenylpropenoyl-L-amino acids, and stilbenes [5,12,13,14,15,16,17,18,19,20].

Polyphenols represent approximately 13% of the dried unfermented cocoa beans [21], where proanthocyanidins can reach 58% of the total polyphenol content, followed by catechins and anthocyanins with 37% and 4%, respectively [22]. Proanthocyanidins are usually (epi) catechin-based, i.e., procyanidins, and the main anthocyanins are cyanidin-3-galactoside and cyanidin-3-arabinoside [21,23,24]. 

The main methylxanthines are theobromine, caffeine, theophylline, and 7-methylxanthine. Theobromine is the most concentrated methylxanthine, and it could be present in concentration levels of 1–2% (dry basis) in cocoa seeds; caffeine also has an important contribution from 0–2% (dry basis) [5].

Additionally, *N*-phenylpropenoyl-L-amino acids have been identified in cocoa and described as polyphenol-amino acid conjugates, some of which have been related to antioxidant mechanisms, inducers of mitochondrial activity and inhibitors of pathogen adhesion in stomach tissues [20].

Cocoa and cocoa derived products suffer significant changes throughout the processing, and this has to be taken into account when studying the effects of cocoa intake on human health.

Traditionally, cocoa undergoes different processing steps after harvesting, some of which are fermentation, drying, roasting [6], and size reduction steps.

During fermentation and drying cocoa beans have extensive proteolysis, oxidation, and polymerization reactions, among others, resulting in a decrease on total flavonoids and the emergence of new metabolites [5,25,26,27,28].

Also, during roasting, high temperatures lead to glucose and sugar depletion, and to the formation of pyrazines, pyrroles, quinoxalines, pyrones, phenylalk-2-enals, lactones, diketopiperazines, and derivatives of phenylalanine, furanones and others. Compounds, such as triacylglycerols, alcohols, esters, and acids, do not go through significant changes [5]. On the other hand, flavanols and procyanidins can be affected by different temperature dependent reactions, as for example epimerization [3,29,30,31].

Roasted beans [3,5] are the starting point for chocolate and cocoa powder production, which are the cocoa derived products most commonly consumed. In both cases, formulation steps are needed, which increase the diversity of compounds present in these matrices and their interactions.

### 1.2. Cocoa and Health: General Aspects

A variety of health benefits have been associated to the intake of cocoa and its derived products, many of them attributed to the intake of polyphenols, particularly flavonoids [32,33,34]: improvement in insulin resistance by lowering serum insulin and in flow-mediated dilatation [35], as well as improvement in blood pressure, maintenance of normal endothelium-dependent vasodilation, vascular and platelet function, increased cerebral blood flow, potential cancer prevention, and anti-inflammatory and antioxidant activity [36,37,38,39,40]. Nonetheless, contradictory evidence has been reported indicating that more research is still needed [41,42,43,44,45,46].

It has been proposed that cocoa polyphenols can also have an influence on the gut microbiota, promoting the development of microorganisms as *Lactobacillus* spp. and *Bifidobacterium* spp. Microbiota can metabolize polyphenols into different bioactives, as valerolactones, phenylpropionic and phenylacetic derivatives some of which could activate anti-inflammatory pathways [33,47,48]. 

Other bioactive compounds, like methylxanthines, have shown possible beneficial effects. For example, caffeine may improve exercise performance, but more research is needed to understand the mechanisms behind this statement [49].

To understand the effects of cocoa on human health, it is fundamental to know which metabolites are produced after cocoa intake, as this can help understand the absorption, distribution, metabolism, and excretion mechanisms [47,50]. The development of different types of clinical trials contributes to this knowledge; and, in relation to cocoa, there is a particular need to promote long term research.

This review collects the results of different studies that traced several metabolites in urine and plasma after the acute or chronic cocoa intake.

## 2. Methodological Considerations

Data collection was done by searching individual and compound keywords in different databases, such as: cocoa, chocolate, humans, randomized, acute, health, untargeted, non-targeted, metabolomics, urine, plasma, pharmacokinetics, polyphenols, (epi) catechin, procyanidin, methylxanthines, and theobromine. The main databases used were: Google Scholar, ScienceDirect, ACS Publications, and Springer Link. The searches were done in between August 2016 and February 2019 and included studies from 1999 to February 2019.

## 3. Results

Table 1 and Table 2 summarize several studies that analyze the effects of the intake of different cocoa derivatives, such as chocolate (Table 1) and cocoa powder drinks or extracts (Table 2) in human plasma and urine samples using a targeted methodology. Meanwhile Table 3 summarizes non-targeted researches, including chocolate or cocoa powder drink intake studies, as well as one cross sectional study.

The methodological aspects are detailed, including the main objective of the studies, the experimental design applied [51,52,53,54], information regarding the subjects, matrices and dose employed, the biological samples studied, the analytical instrumental technique used to measure the metabolites, and the statistics applied to process data.

A total of 34 studies were reviewed, 30 of them used a targeted approach, and 4 of them were untargeted. The clinical studies employed different cocoa derived matrices. Nine of these studies included chocolate intake intervention, 24 used cocoa powders or cocoa extracts beverages, and 2 of them had a cross-sectional experimental design which did not apply dietary interventions [55,56].

Most researches followed acute single dose interventions, nonetheless only 2 of them evaluated the effect of a daily cocoa-derived products intake after 4 weeks [57,58]. Additionally, 10 studies included a non-cocoa-derived control intake in their experimental design.

Regarding the type of samples analyzed, most studies (*n*) investigated plasma (*n* = 8), urine (*n* = 14) or both plasma and urine (*n* = 12). Additionally, one study analyzed saliva [59] and another studied feces [60].

The number of volunteers (n) that finalized clinical studies was varied. In five studies only 5 volunteers participated, in ten studies 5 < *n* ≤ 10 were part of the clinical trial, eight studies included 10 < *n* ≤ 20 and twelve studies recruited and selected volunteers in a range from 21 to 80 volunteers.

Most clinical studies investigated samples from healthy non-smokers. Only two trials obtained samples from smokers [61] and from volunteers with high cardiovascular risk [57]. Ten studies investigated only men, 18 studies men and women, and one study investigated children [56].

LC-UV/Vis (liquid chromatography couple to ultraviolet or visible detectors) and FLD (fluorescence detectors), and LC-MS (liquid chromatography couple to mass spectrometers) were the predominant analytical platforms reported. In particular, when using an untargeted approach, LC-MS was present in all studies; and one study also applied NMR (nuclear magnetic resonance) [20]. 

Further on, Table 4, Table 5, Table 6, Table 7 and Table 8 were used to classify the metabolites identified in urine and plasma, in different studies with different approaches. 

In particular, studies that mentioned acute intake of cocoa are summarized in the following tables: (a) Table 4 and Table 6 include information of different metabolites as concentrations measured in the biological samples and the corresponding sampling periods. The type of matrix analyzed is also indicated. Both tables summarize information of targeted studies, the first focused on plasma (Table 4) and the other in urine (Table 6) analysis; (b) Table 5 shows the time after intake of different chocolates, where the highest signals of the different metabolites were detected or expected in plasma samples, (c) Table 7 focuses on the main metabolites found in urine that discriminated between different periods of time after intake, with an untargeted approach.

On the other hand, discriminating metabolites in between regular/chronic consumers and non-regular/chronic consumers observed in urine and plasma are listed on Table 8. This covers both targeted and untargeted studies.

Metabolites were organized by means of the chemical group, mainly methylxanthines, flavonoids and conjugates, phenolic acids, and lignans. As for the data collected from untargeted studies, data organization was done in a way that respected as much as possible the authors´ original classification. Additionally, in each category metabolites were organized alphabetically.

## 4. Discussion

### 4.1. Methylxanthines 

Theobromine showed some of the higher concentrations in plasma and urine after cocoa intake in different human intervention trials (Table 4 and Table 5). One study [81] showed that unmetabolized theobromine in urine was close to 1.5 times higher when consuming methylxanthine enriched powder compared to the non-enriched cocoa powder, which indicates that renal clearance could vary in function of exposure. Due to a possible saturation of the pathway, a lack of enzymes and/or cofactors to process the metabolite may have had affected [81,88].

There is evidence that suggests that excretion also depends on gender and age. After consumption of chocolate in a cross-over study, theobromine excretion showed by women almost doubled man´s levels and was also higher for volunteers younger than 29 years, and lower compared to the ones older than 54 years [84].

A variety of methylxanthines metabolites were also observed in non-targeted studies (Table 7), which follow characteristic metabolic pathways [89]. Some of them have been proposed as possible biomarkers of regular cocoa intake as AMMU, 3-methyluric acid, 7-methylxanthine, 3-methylxanthine, theobromine, and 3,7-dimethyluric acid [55].

### 4.2. Polyphenols

Polyphenols such as (epi) catechin, and the corresponding glucuronides, sulfates, with or without methylations were the most frequently analyzed metabolites. 

One example is a study that evaluated plasma and urine samples after drinking a cocoa beverage. The most abundant flavanol metabolites were (-)-epicatechin-3´-β-D-glucuronide, (-)-epicatechin-3´-sulfate, 3´-*O*-methyl-(-)-epicatechin-5-sulfate, and 3´-*O*-methyl-(-)-epicatechin-7-sulfate which represented close to 94% of the total flavanol metabolites that were measured in plasma [82].

This is consequence of the metabolic pathways that flavanols and phenolic acids follow. When absorbed, these metabolites are transferred to the liver or other tissues where phase I and phase II reactions occur. In phase I, cytochrome enzymes lead to hydroxylations, oxidations and reductions; meanwhile, phase II produces conjugates by action of glucuronosyl transferases, sulfotransferases, and catechol-*O*-methyl transferases [75,90,91,92].

In general terms these metabolites reached maximum concentrations in plasma in less than 4 hours after intake (Table 4). One exception was (-)-epicatechin-7´-β-D-glucuronide, that in one study required 12.8 ± 4.8 h after cocoa intake to reach maximal concentrations [66].

When these metabolites are present in plasma, they could exert particular effects. For example, one study analyzed the impact of chocolate intake on oxidative damage, and observed an increase in plasma antioxidant activity and plasma lipid oxidation products decrease as epicatechin plasma levels increased [63]. Additionally, elimination rates have an influence on the probabilities of accumulation in blood or tissues, and the subsequent effect on health [93].

In this matter, when studying urine samples, the required time to reach maximal concentration was found to be largely variable. While (epi) catechin-*O*-sulfate isomer required a time in between 0–2 h after cocoa powder in milk intake to reach the higher concentration [77], (-)-epicatechin-7-β-D-glucuronide required a time in between 10–24 h after dark chocolate consumption [66].

Influence of age on metabolism was analyzed in an intervention that evaluated two cocoa powder drinks. Non-significant differences or small differences were observed for the different pharmacokinetic parameters, in the renal clearance, total excreted metabolites, and apparent volume of distribution in two age groups: 18–35 years and 65–80 years. Some of the small differences observed were associated with changes in renal function due to normal aging processes [82].

Studies converge in that increasing polymerization degree (*n* > 2) of procyanidins hinders their absorption. Procyanidin B2 dimer has been detected in plasma after consuming a cocoa beverage, showing a maximum concentration in the period between 0.5–2 h (Table 5). In this same study, B5 dimer was not detected [68]. Similar results were observed in another study where volunteers drank samples with cocoa extracts that contained procyanidins from 2–10 units. In this study, B2 dimer was detected in plasma in approximately 80% of the volunteers, indicating maximum concentrations at 2 h after intake, and with concentrations close to the limit of detection. Additionally, B5 dimer was not detected in any of the plasma samples [80]. 

Polyphenol absorption and excretion can depend on the food matrix, but contradictory information exists. For example, excretion patterns of microbial phenolic acids metabolites measured in urine differed depending on the liquid vehicle of cocoa powder consumed: water or milk [78]. One randomized study compared the levels of (-)-epicatechin glucuronide in plasma after the intake of cocoa powder with milk or water, and reported no statistical differences in the results from each group [73]. Also, milk protein did not affect the bioavailability of polyphenols present in a chocolate drink [74]. In another study the excretion of different flavan-3-ol metabolites within 24 h after cocoa intake was close to 20% of the ingested dose if cocoa was provided as a water-based beverage, but only 10% if ingested as a milk-based cocoa drink [77]. When consuming two cocoa beverages, either prepared with water or with milk, the total excretion was not affected, but kinetics was different. In the same period of time after intake (-)-epicatechin glucuronide was the most concentrated metabolite when water was the vehicle—doubling the concentration of total excreted (-)-epicatechin sulfates- but when milk was used, glucuronides and total sulfates reached similar concentrations in urine [75]. No significant differences in excretion patterns were found when comparing a variety of metabolites in urine after consuming cocoa powder in milk or in water [86]. It is suggested that polyphenol absorption and metabolism might be affected because of the interactions between milk polypeptides and polyphenols, which can decrease the bioaccessibility [94,95]. In addition, polyphenol absorption could also be affected by carbohydrates, as it has been reported that flavanol uptake could increase when simultaneously consuming these macronutrients [50].

Flavanol absorption and metabolism has also been demonstrated to be dependent on the stereochemistry. Differences in absorption and metabolism were detected after the consumption of equal amounts of (-)-epicatechin, (-)-catechin, (+)-catechin, and (+)-epicatechin in a cocoa based milk drink [79].

Non-absorbed flavonoids and phenolic acids could follow metabolic pathways that are carried out by the colonic microbiota [66,90,91,92]. Some epicatechin metabolites can return to small intestine including non-absorbed epicatechin pass to the colon, which can account a total close to 70 % of the total ingested [96].

Microbial metabolism can lead to the production of phenolic acids, sulfated and glucuronidated valerolactones [57]. Some examples are ferulic acid, phenylacetic acid, hippuric acid (Table 6), 4-hydroxy-5-(dihydroxyphenyl)-valeric acid glucuronide, and methoxyhydroxyphenyl valerolactone (Table 6 and Table 7).

In general terms, these types of metabolites require longer times to reach mean concentrations in urine and plasma than the metabolites following phase II metabolism (Table 4 and Table 6, Table 7 and Table 8). To illustrate, ferulic and *m*-hydroxyphenyl acetic acids showed maximum concentrations in between 24 and 48 h after chocolate intake, and vanillic acid was the only metabolite that returned to basal level concentrations in the 48 h of observation [65].

Research suggests that consumption of cocoa and derivatives could modulate human metabolism. A randomized controlled cross-over intervention trial analyzed the acute consumption of three types of chocolates in 42 healthy volunteers and concluded that several endogenous metabolites were affected (Table 5 and Table 7). In particular, several amino acids, organic acids, creatinine, lactate, and N^1^-methylnicotinamide decreased meanwhile pyruvate, tyrosine, and *p*-hydroxyphenylacetate increased after the intervention. Flavan-3-ols, methylxanthines and their metabolites were suggested to modulate endogenous and colonic microbial metabolism [84].

Studies with longer periods of intervention detected additional metabolome changes due to regular cocoa consumption (Table 8). For example, different glucuronide and sulfate conjugates of (-)-epicatechin, *O*-methyl-epicatechin, dihydroxyphenyl valerolactones, and methoxy hydroxyphenyl valerolactones increased their levels in plasma after regular consumption of cocoa powder in a non-healthy group of volunteers. Hydroxyphenylacetic acids, AMMU isomers, 5-(3´,4´-dihydroxyphenyl)-γ-valerolactone, and its glucuronides and sulfates have been proposed as biomarkers of regular consumption of cocoa [55,57]. Further on, one study reported that methylglutaryl carnitine, derived from the acylcarnitine pathway, decreased after a long-term cocoa intake [55].

More recent studies have supported this information as 5-(3´,4´-dihidroxyphenyl)-γ-valerolactone has also been proposed as a specific biomarker of flavan-3-ols consumption, deriving only from (epi) catechin-based mono and oligomeric cocoa flavanols [83].

There is also evidence that polyphenol consumption from cocoa products might change the gut microbiota, exerting prebiotic effects, and which could be related to the activation of anti-inflammatory pathways with benefits in the host and alter the obtained profile of metabolites. One study observed an increase of *Lactobacillus* spp. and *Bifidobacterium* spp. in contrast with a decrease of *Clostridium* spp. populations after daily consumption during one month of a flavanol-rich cocoa beverage, compared to a low flavanol cocoa beverage [33,97,98].

### 4.3. Other Metabolites

Metabolites different than methylxanthines and polyphenols metabolism have also been detected after cocoa intake. For example, trigonelline (*N*-methylnicotinic acid) and hydroxynicotinic acid may be a result of nicotinic acid metabolism. They were detected in urine only within 6 h after cocoa powder consumption. Also, diketopiperazines such as cyclo(ser-tyr) and cyclo(pro-pro) were reported for the first time associated to cocoa powder intake and are considered flavor and taste metabolites [86].

*N*-phenylpropenoyl-L-amino acids have also been metabolites of interest as there is some preliminary evidence of their biological activity. Thirteen of them have been reported in urine after cocoa intake [20].

Additionally, significant changes in plasma metabolites after 6 h of chocolate intake have been reported for β-hydroxybutyrate, acetone, acetoacetate, and aspartate. (Table 5) [84].

One study observed that daily dark chocolate intake for 2 weeks modulated energy and hormone metabolism of humans with low and high anxiety traits, and also modulated their microbial gut metabolism. Urine analysis showed a reduction of stress hormone metabolites, and other stress-related metabolites presented a trend to change towards the low anxiety profile. Some of these metabolites are glycine, citrate, trans-aconitate, proline, DOPA, β-alanine, hippurate, and *p*-cresol sulfate [99]. This can eventually help understand the impact of cocoa intake on health.

## 5. Conclusions

A diversity of metabolites has been identified in urine and plasma after consumption of cocoa or cocoa derived products. Theobromine and caffeine have been some of the main methylxanthines identified together with their metabolites. Polyphenolic phase I and phase II metabolism leads mainly to (epi)catechin sulphates, glucuronides, and sulfoglucuronides. Non absorbed polyphenols are transformed by colonic microorganisms yielding a diversity of metabolites that range from phenolic acids to different valerolactones, and more complex metabolites.

Biomarkers of consumption of cocoa have been proposed: AMMU, 3-methyluric acid, 7-methylxanthine, 3-methylxanthine, theobromine, 3,7-dimethyluric acid, hydroxyphenylacetic acid, and 5-(3´,4´-dihydroxyphenyl)-γ-valerolactone; the latter with its correspondant glucuronides and sulfates.

Evidence shows that absorption, metabolism, and excretion of cocoa metabolites depend on the food matrix, the dose, age, gender, overall health status and other factors such as the polymerization degree (e.g., procyanidins), and stereochemistry (e.g., flavanols).

The development of clinical studies is fundamental to understand the metabolic pathways of different metabolites present in cocoa and further on the effects on health. Special attention must be given to properly design the experiments, and instrumental methodologies for the extraction and quantification of metabolites in biological samples. 

## Figures and Tables

**Table 1:** Summary of the main aspects of targeted cocoa studies, involving chocolate intake.

Objectives	Experimental Design	Subjects	Matrices Description and Dose	Biological Samples	Sampling period	Analytical Technique	Statistical Analysis	Year	Reference
Analyze plasma kinetics of epicatechin after dark chocolate intake.	Non-randomized cross-over. 1-week washout.	8 males. Average age and BMI of 40 years and 23.9 kg/m^2^.	40 g and 80 g of dark chocolate with bread.	Plasma.	0, 1, 2, 3, 4, and 8 h.	HPLC-UV or fluorescence.	Student´s and Wilcoxon tests.	1999	[62]
Evaluate changes in plasma epicatechin levels, antioxidant capacity and plasma lipid oxidation products after chocolate intake.	Randomized, cross-over. 1-week washout.	14 women and 6 men. From 20 to 56 years, with average BMI of 23.8 kg/m^2^.	0, 27, 53, and 80 g of semi-sweet chocolate rich on procyanidins, and 47 g of bread.	Plasma.	0, 2, and 6 h.	HPLC coupled with a coulometric detector.	ANOVA with control for multiple measurements, and Tukey–Kramer test.	2000	[63]
Evaluate (-)-epicatechin and its metabolites in plasma and urine after cocoa and chocolate intervention.	Cross-over. 6-day washout.	5 males. Ages from 30 to 33 years and BMI from 20.4 to 23.9 kg/m^2^.	Chocolate or cocoa.	Urine and plasma.	Plasma: Baseline, 1, 2, 4, 8, and 24 h.Urine: 0–8 h, 8–24 h.	HPLC and LC-MS negative mode.	Student´s *t*-test.	2000	[64]
Quantify in urine, different phenolic acids formed in the colon after the consumption of chocolate.	Uncontrolled.	7 men and 4 women. Mean age of 24, mean height of 172 cm and mean weight of 67 kg.	80 g of chocolate.	Urine.	Baseline, 0–3, 3–6,6–9, 9–24, and 24–48 h.	GC-MS, HPLC-DAD, HPLC-ESI-MS in negative ionization mode.	ANOVA and Tukey test.	2003	[65]
Determine theobromine and caffeine in saliva, plasma and urine after dark chocolate intake.	Uncontrolled.	5 healthy volunteers with no dietary restrictions.	41 g of dark chocolate bars.	Urine, saliva, and plasma.	Baseline and 90 min.	UPLC-DAD triple quadrupole MS/MS.	No tests were applied. Data was expressed as averages with confidence intervals.	2010	[59]
Identify and quantify (-)-epicatechin conjugates in plasma and urine after chocolate intake.	Uncontrolled.	5 healthy volunteers. Average age of 23 years and average BMI of 22 kg/m^2^.	100 g of 70% chocolate having 79 mg of (-)-epicatechin, 26 mg of (±)-catechin, and 49 mg of procyanidin B2.	Urine and plasma.	Plasma: Baseline, 15, 30, 45 min, and 1, 1.25, 1.5, 1.75, 2, 2.25, 2.5, 3, 4, 6, 8, 10, 14, 18, and 24 h. Urine: Baseline, 0–5 h, 5–10 h, 10–24 h, and 0–24 h.	UPLC-ESI-Quattro Micro API.	One-way ANOVA with Tukey test.	2012	[66]
Analyze the relation between chocolate consumption, glucose metabolism, and exercise performance.	Single blinded, randomized, cross-over.	16 male cyclists (from 4 to 20 h/week of training).	Cocoa enriched dark 70% chocolate and cocoa depleted control.	Plasma.	−10, 0, 15, 30, 45, 60, 90, and 120, 140, 180, 210, 240, 300, and 360 min.	HPLC-UV/Vis	Mixed model with F-test, Hidges-Lehmann, Wilcoxonsigned-rank and Student´s *t*-test.	2014	[49]
Evaluate the effects of intake of milk chocolate, cocoa powder or dark chocolate consumption on uric acid crystallization.	Cross-over.	11 males and 9 females with ages from 22 to 65 years.	2 × 20 g of a cocoa derived product/day (milk chocolate, chocolate powder and dark chocolate).	Urine.	Baseline and urine obtained after 12 h overnight at the end of the intervention.	HPLC-UV/Vis.	ANOVA, Bonferroni and Wilcoxon signed-rank tests.	2018	[67]

**Notes:** The abbreviation BMI corresponds to body mass index, and BW to body weight.

**Table 2:** Summary of the main aspects of targeted cocoa studies, involving cocoa powder or cocoa extracts-based drinks intake.

Objectives	Experimental Design	Subjects	Matrices Description and Dose	Biological Samples	Sampling period	Analytical Technique	Statistical Analysis	Year	Reference
Determine the presence of specific procyanidins in human plasma after consumption of cocoa flavanol extract.	Uncontrolled.	3 men and 2 women. Age: 23–34 years, average body weight 70.5 kg.	0.375 g of cocoa extract/kg BW in 300 mL of water (average of 26.4 g of cocoa).	Plasma.	Baseline, 0.5, 2 and 6 h.	HPLC-coulometric electrochemical multiple-array detection; HPLC-MS.	Kruskal–Wallis one-way ANOVA and Tukey or Dunn tests.	2002	[68]
Evaluate the effect of a flavanol-rich cocoa beverage on the circulating pool of nitric oxide and of endothelial dysfunction.	Randomized, double-blinded, cross-over.	6 males and 5 females, with mean age of 31, and mean BMI of 21.8 kg/m^2^. They smoked an average of 17 cigarettes/day.	High and low flavanol content cocoa drink in water.	Plasma.	Baseline and 2 h.	HPLC-FLD.	ANOVA, pairwise tests with Bonferroni correction for multiple comparisons.	2005	[61]
Measure different metabolites in urine after polyphenol-rich beverages intake, with a fast method.	Randomized, cross-over. 14-day washout.	5 women and 4 men, with ages in between 20–32 years and BMI in between 18.9–24.8 kg/m^2^.	10 g of cocoa powder in 200 mL of water (other non-cocoa beverages were tested in the research), or hot water.	Urine.	0–24 h.	HPLC-ESI-MS/MS	Mann–Whitney *U* test.	2005	[69]
Developing a rapid and reproducible method for analysis of (-)-epicatechin metabolites in plasma and urine.	Randomized, cross-over.	2 women and 3 men in a range of 18–49 years.	250 mL of milk or 40 g of cocoa powder in 250 mL of milk.	Plasma and urine.	Plasma: 0 and 2 h Urine: 0 and 6 h.	HPLC coupled to an API-QQQ-MS.	Student´s *t*-test.	2005	[70]
Evaluate if dietary flavanols and their metabolites can function as vasoactive mediators.	Randomized, double-blinded, cross-over. Minimum 2-day washout.	16 males within 25–32 years and with a BMI of 19–23 kg/m^2^.	High and low flavanol content cocoa powder with 300 mL of water.	Plasma.	Baseline, 1, 2, 3, 4, and 6 h.	HPLC-MS	ANOVA, pairwise tests with Bonferroni correction for multiple comparisons.	2006	[71]
Analyze (-)-epicatechin metabolites and total antioxidant activity after cocoa beverage intake.	Randomized and cross-over.	9 women and 12 men, within 18 and 50 years, and with a BMI of 19.1–27.7 kg/m^2^.	40 g of cocoa powder with 250 mL of water and 250 mL of milk as control.	Urine.	Baseline, 0, 6, 12, and 24 h.	API-QQQ-MS/MS.	ANCOVA and Student´s *t*-test; Pearson´s correlation.	2007	[72]
Analyze the effect of milk in the bioavailability of (-)-epicatechin from a cocoa powder.	Randomized and cross-over. 1-week washout.	9 women, 12 men, within 18 and 50, and with a BMI of 19.1–27.7 kg/m^2^.	250 mL of milk used as a control, 40 g of cocoa powder dissolved in 250 mL of water or milk.	Plasma.	Baseline, 2 and 6 h.	LC-MS.	ANCOVA.	2007	[73]
Determine the effect of milk protein addition on the uptake of cocoa polyphenols by analyzing metabolites in plasma samples, after a cocoa drink intervention.	Randomized, controlled, double blind, and cross-over. 1-week washout.	10 men and 14 women. Age in the range of 52–65 years with BMI in the range of 18–30 kg/m^2^. 13 volunteers were taking medications or dietary supplements.	200 mL of dairy and non-dairy chocolate drink.	Plasma.	Baseline, 0.5, 1, 1.5, 2, 3, 4, 6, and 8 h.	Analytical: HPLC-fluorescence.	Paired *t*-test with Bonferroni correction for multiple tests.	2007	[74]
Quantify and evaluate human metabolism of *N*-phenylpropenoyl-L-amino acids present in a cocoa drink.	Uncontrolled.	4 males and 4 females, from 24 to 30 years.	Cocoa powder-based beverage.	Urine.	Baseline, 1, 2, 3, 4, 6 and 8 h.	LC-MS/MS multiple reaction monitoring and NMR.	Not specified.	2008	[20]
Evaluate the impact of milk addition on the (-)-epicatechin metabolites after intake of a cocoa drink.	Randomized cross-over. 1-week washout.	9 women and 12 men with ages between 18–50 and BMI from 19.1 to 27.7 kg/m^2^.	Cocoa beverage with 40 g of cocoa powder and: (a) 250 mL of milk; (b) 250 mL of water or (c) 250 mL of milk without cocoa powder.	Urine.	0–6, 6–12 and 12–24 h.	HPLC coupled to an API-QQQ-MS.	ANCOVA and Student´s *t*-test.	2008	[75]
Develop an analytical method for determining cocoa metabolites in human and rat urine.	Uncontrolled.	9 women and 12 men, within 18 and 50 years and a mean BMI of 21.6 kg/m^2^.	40 g of cocoa powder in 250 mL of water.	Urine.	Baseline and 24 h after intake.	SPE and LC-MS/MS.	Wilcoxon test.	2009	[76]
Determine the effect of milk on the bioavailability of cocoa flavan-3-ol metabolites by evaluating plasma and urine samples after cocoa powder intervention.	Controlled, cross-over. 4-week washout.	6 man and 3 women. Ages in the range of 20–43. Average BMI of 24.7 kg/m^2^.	10 g of cocoa powder in 250 mL of milk or water with 1 g of paracetamol and 5 g of lactulose.	Urine and plasma.	Plasma: baseline 0.5, 1, 2, 3, 4, 6, 8, and 24 h. Urine: baseline, 0–2, 2–5, 5–8 and 8–24 h.	HPLC-PDA-MS^2^.	2-factor repeated measures ANOVA and Student´s *t*-test.	2009	[77]
Evaluate plasma and urine metabolites after cocoa powder intake in high cardiovascular risk subjects.	Randomized, controlled and cross-over.	High CVD risk patients: 19 men and 23 women. Average age of 69.7 years.	2 × 20 g of cocoa powder/day with 250 skimmed milk or only 500 mL/day of skimmed milk for 4 weeks.	Urine and plasma.	0–24 h.	LC-MS/MS.	Wilcoxon test.	2009	[57]
Evaluate the effect of milk on the urinary excretion of colonic microbial-derived phenolic acids after cocoa powder intake.	Randomized and cross-over.	12 men and 9 women. Age in the range of 18–50 years with BMI of 21.6 kg/m^2^.	40 g of cocoa powder in 250 mL of water or milk.	Urine.	Baseline, 0–6, 6–12, and 12–24 h.	LC-MS/MS and LC-PAD.	Wilcoxon test for related samples.	2010	[78]
Study the stereochemical configuration of four different flavanols on their absorption, metabolism, and biological activity.	Randomized, double-blinded, cross-over.	7 males within 18 and 35 years old, with average BMI of 24 kg/m^2^.	1.5 mg/kg BW of (-)-epicatechin, (+)-epicatechin, (+)-catechin, (-)-catechin, with 0.5 g/kg BW of low-flavanol cocoa based dairy drink.	Urine and plasma.	Urine: collected over 24 h. Plasma:baseline, 2 and 4 h.	HPLC-UV/VisAnd fluorescence.	Two-way repeated measures ANOVA and Tukey´s test.	2011	[79]
Quantify different metabolites in plasma and urine, after the consumption of a cocoa drink with added flavanols and procyanidins.	Randomized, double-masked, cross-over.	12 males in between 18 and 35 years old, with average BMI of 24 kg/m^2^.	0.48 g/kg BW of flavanol (F) and procyanidin (P) free mimetic cocoa drink powder reconstituted in 4 g/kg of BW of milk with 1% fat with (a) cocoa extract with monomeric F and P, (b) cocoa extract high in P or (c) (-)-epicatechin isolated from cocoa.	Urine and plasma.	Urine: 0–7 h and 7–24 h. Plasma: Baseline, 1, 2, and 4 h.	HPLC-UV and coulometric electrochemical detector, and HPLC-diode array detector.	Two-way repeated measures ANOVA and Tukey´s test.	2012	[80]
Study the bioavailability of methylxanthines in two soluble cocoa products by evaluating plasma and urine samples the after intervention.	Randomized, controlled, single-blind, cross-over. 10-day washout.	3 males and 10 females. Ages in the range of 18–45 years. Average BMI of 22.5 kg/m^2^ for men and 23.4 kg/m^2^ for women.	15 g of the powder without enrichment and 25 g of methylxanthine-enriched powder, with 200 mL of semi-skimmed milk.	Urine and plasma.	Plasma: baseline, 0.5, 1, 2, 3, 4, 6, and 8 h. Urine: baseline, 0–4, 4–8, 8–12, and 12–24 h.	HPLC-DAD, HPLC-Q-ToF in positive ionization mode.	Mixed model.	2014	[81]
Analyze the bioavailability, metabolism and microbial breakdown of (-)-epicatechin, procyanidin B1, and a cocoa procyanidin fraction.	Randomized, double-blinded, cross-over. 1-week washout.	7 healthy male volunteers with ages in between 24 and 31 years and with a BMI of 22 and 26 kg/m^2^.	Pure (-)-epicatechin, pure procyanidin B1 and a purified cocoa polymeric procyanidin fraction.	Plasma, urine and feces.	Plasma: baseline, 1, 2, 4, 8, 24 and 48 h. Urine: 0–4, 4–8, 8–24 h, and 48 h. Feces: 0–24 h.	GC-MS/MS and HPLC-ESI-Q-MS.	Not specified.	2015	[60]
Validate and HPLC method for measuring theobromine in urine and evaluating theobromine urinary levels in children with different cocoa intake patterns.	Cross-sectional.	80 healthy children from 8–17 years: 26 did not consume cocoa derived products, 19 of them did not consumed cocoa powder but did consume 1 cocoa derived product daily, 12 children just consumed cocoa powder in breakfast and 23 consumed cocoa derived products > once a day.	No dietary intervention or recommendations.	Urine.	12 h in the day and 12 h in the night.	HPLC-UV.	Kruskal–Wallis and Wilcoxon signed-rank tests.	2015	[56]
Compare absorption, metabolism, and excretion after a cocoa drink intake.	Randomized cross-over. 1-week washout.	40 males. Group 1 (young): 18–35 years and average BMI of 24 kg/m^2^; Group 2 (elderly): 65–80 years and average BMI of 27 kg/m^2^.	Fruit-flavored cocoa powder drinks with 5.3 mg or 10.7 mg of cocoa flavanols/kg BW.	Plasma and urine.	Plasma: 0, 0.5, 1, 1.5, 2, 3, 4, 5, 6, and 24-h. Urine: 0–2, 2–4, 4–6, 6–12, 12–24 h.	HPLC-FLD/UV and electrochemical detection, and HPLC-UV.	Two-way ANOVA with Bonferroni and Tukey tests.	2015	[82]
Evaluate the effects of milkand dark chocolate and cocoa powder intake on uric acid crystallization.	Cross-over.	11 males and 9 females with ages from 22 to 65 years.	2 × 20 g/day of a cocoa derived product: milk chocolate, chocolate powder and dark chocolate.	Urine.	Baseline and urine obtained after 12 h overnight at the end of the intervention.	HPLC-UV/Vis.	ANOVA, Bonferroni and Wilcoxon signed-rank test.	2018	[67]
Analyze the use of gVL-3´/4´-sulphate and gVL-3´/4´-*O*-glucuronide as biomarkers of flavan-3-ols intake.	Randomized, double-masked, cross-over.	I. 8 males, from 25–60 years. II. 14 males, from 25–40 years.	I. 8 different nondairy drinks with flavan-3-ols (F) with 34.8 mg of (-)-epicatechin or the equivalent concentration of one of the following: (-)-epigallocatechin, (-)-epicatechin-3-*O*-gallate, (-)-epigallocatechin-3-*O*-gallate (isolated from green tea), 1:1:1 of theaflavin-3-*O*-gallate, theaflavin-3‘-*O*-gallate and theaflavin-3,3‘-*O*-digallate, thearubigins, (isolated from black tea), procyanidin B2 isolated from cocoa, and a control without added F. II. Four different nondairy drinks with different amounts of flavan-3-ols ranging from 95 mg to 1424 mg per volunteer.	Urine.	I. 0–6 and 6–24 h. II. 0–4, 4–8, 8–12, and 12–24 h.	UPLC-MS.	ANOVA and Student‘s *t*-test.	2018	[83]
Evaluate the effect of cocoa intake on lipid profiles and biomarkers of oxidative stress, and arachidonic acid/ eicosapentaenoic acid ratio.	Randomized, three-arm parallel group.	48 healthy subjects divided in three groups: low-cocoa (*n* = 16), middle-cocoa (*n* = 16), and high-cocoa group (*n* = 16). Average age and BMI were close to 44 years and 23 kg/m^2^.	Cocoa capsules dosed to different groups: a) low-cocoa group: 1 g of cocoa (55 mg/day), middle-cocoa group: 2 g of cocoa (110 mg flavanols/day), and high-cocoa group: 4 g of cocoa (220 mg flavanols/day), for 4 weeks.	Plasma and urine.	0, 1, 2, and 4 (at the beginning of the study and after 4 weeks of intervention).	HPLC-FLD-UV	ANOVA and Tukey´s test.	2018	[58]

**Notes:** The abbreviation BMI corresponds to body mass index, and BW to body weight.

**Table 3:** Summary of the main aspects of untargeted cocoa intervention studies.

Objectives	Experimental Design	Subjects	Matrices and Dose	Biological Samples	Sampling period	Analytical Technique	Statistical Analysis	Year	Reference
Study the chemical profile of plasma and urine after flavan-3-ol enriched dark chocolate, standard dark chocolate and white chocolate intake.	Randomized, controlled and cross-over. Minimum 2-wk washout.	16 males and 26 females with average BMI of 24.5 kg/m^2^.	60 g of: flavan-3-ol (FLA) enriched dark chocolate, standard dark chocolate low in FLA and white chocolate with no FLA.	Plasma and urine.	Baseline, 2 and 6 h. (1)	1H NMR (600 MHz) and HPLC-ToF-MS.	PCA and PLS-DA. Kruskal–Wallis and Dunn tests.	2017, 2013	[84,85]
Evaluate the changes in humane urine metabolome after cocoa powder intake.	Randomized, controlled and cross-over. 1-week washout.	5 men and 5 women. Ages in the range of 18–50 and average BMI of 21.6 kg/m^2^.	40 g of cocoa powder in 250 mL of water or milk and 250 mL of milk as a control.	Urine.	Baseline, 0–6, 6–12 and 12–24 h.	HPLC-Q-ToF. Positive ionization mode.	PCA, PLS-DA, (OSC) PLS and OSC-PLS-DA.	2009	[86]
Analyze the changes in urine metabolome after cocoa powder intervention.	Uncontrolled.	5 women and 5 men between 18–50 years with BMI 21.6 kg/m^2^.	40 g of cocoa powder with 250 mL of milk.	Urine.	Baseline, 0–6, 6–12, and 12–24 h.	HPLC-Q-ToF-MS in positive mode.	PLS-DA, OSC-PLS-DA and two-way hierarchical clustering (HCA) applying Bonferroni correction.	2010	[87]
Apply an untargeted metabolomics approach to propose a model that can discriminate the urinary metabolome of regular cocoa product consumers and non-consumers, revealing dietary biomarkers, in a free-living population.	Randomized, controlled, parallel group, multicenter, and cross-sectional.	64 high risk (2) free-living subjects. 32 were classified as cocoa consumers (at least 3 servings/week of chocolate and/or cocoa powder) and 32 as non-cocoa consumers (0 g/day) (3).	No dietary interventions or recommendations.	Urine.	Baseline.	Analytical: HPLC-Q-ToF-MS. Positive and negative ionization modes.	OSC-PLS-DA, Mann–Whitney and Student´s *t*-test.	2015	[55]

**Notes:** (1) Samples taken 2 h after intake were analyzed only by NMR, and not by LC-MS. (2) At least with type 2 diabetes mellitus or with at least three conventional cardiovascular risk factors. (3) Baseline data and urine samples were obtained from a PREDIMED study of 275 volunteers.

**Table 4:** Metabolites analyzed in plasma after different acute cocoa intake interventions, by targeted methodologies.

Chemical Category	Metabolite	Treatment 1			Treatment 2			Reference
		Type of Matrix	Concentration	Sampling Period (h)	Type of Matrix	Concentration	Sampling Period (h)	
Flavonoids and conjugates	(-)-epicatechin 3´-sulfate	N.R.	N.R.	N.R.	Dark chocolate	233 ± 60 nmol/L	3.2 ± 0.2	[66]
		Drink with 5.3 mg cocoa flavanols/kg body weight (young)	98 ± 12 nmol/L	1.8 ± 0.2	Drink with 5.3 mg cocoa flavanols/kg body weight (elderly)	125 ± 13 nmol/L	1.7 ± 0.2	[82]
		Drink with 10.7 mg cocoa flavanols/kg body weight (young)	251 ± 20 nmol/L	1.5 ± 0.1	Drink with 10.7 mg cocoa flavanols/kg body weight (elderly)	212 ± 13 nmol/L	1.7 ± 0.1	[82]
	(-)-epicatechin 4´-sulfate	N.R.	N.R.	N.R.	Dark chocolate	11 ± 3 nmol/L	3.5 ± 0.3	[66]
	(-)-epicatechin-3´-β-D-glucuronide	N.R.	N.R.	N.R.	Dark chocolate	290 ± 49 nmol/L	3.2 ± 0.2	[66]
		Drink with 5.3 mg cocoa flavanols/kg body weight (young)	309 ± 41 nmol/L	1.1 ± 0.1	Drink with 5.3 mg cocoa flavanols/kg body weight (elderly)	371 ± 34 nmol/L	1.3 ± 0.1	[82]
		Drink with 10.7 mg cocoa flavanols/kg body weight (young)	551 ± 67 nmol/L	1.2 ± 0.1	Drink with 10.7 mg cocoa flavanols/kg body weight (elderly)	645 ± 66 nmol/L	1.2 ± 0.1	[82]
	(-)-epicatechin-4´-β-D-glucuronide	N.R.	N.R.	N.R.	Dark chocolate	44 ± 11 nmol/L	3.4 ± 0.3	[66]
	(-)-epicatechin-7´-β-D-glucuronide	N.R.	N.R.	N.R.	Dark chocolate	22 ± 6 nmol/L	12.8 ± 4.8	[66]
	(-)-epicatechin glucuronide	Cocoa powder with water	330 ± 156 nmol/L	2	Cocoa powder with milk	273 ± 138 nmol/L	2	[73]
	(4-hydroxyphenyl) acetic acid	N.R.	N.R.	N.R.	Cocoa polymeric procyanidin concentrate	120 ± 40 ng/mL	2 ± 1	[60]
	epicatechin-*O*-sulfate	Cocoa powder with water	83 ± 8 nmol/L	1.4 ± 0.2	Cocoa powder with milk	77 ± 14 nmol/L	1.3 ± 0.2	[77]
	3´-*O*-methyl-(-)-epicatechin 4´-sulfate	N.R.	N.R.	N.R.	Dark chocolate	49 ± 14 nmol/L	3.6 ± 0.3	[66]
	3´-*O*-methyl-(-)-epicatechin 7-sulfate	N.R.	N.R.	N.R.	Dark chocolate	40 ± 10 nmol/L	3.8 ± 0.2	[66]
		Drink with 5.3 mg cocoa flavanols/kg body weight (young)	76 ± 6 nmol/L	1.7 ± 0.2	Drink with 5.3 mg cocoa flavanols/kg body weight (elderly)	63 ± 6 nmol/L	1.8 ± 0.1	[82]
		Drink with 10.7 mg cocoa flavanols/kg body weight (young)	167 ± 19 nmol/L	1.7 ± 0.1	Drink with 10.7 mg cocoa flavanols/kg body weight (elderly)	109 ± 11 nmol/L	1.9 ± 0.2	[82]
Flavonoids and conjugates	3´-*O*-methyl-(-)-epicatechin 5-sulfate	N.R.	N.R.	N.R.	Dark chocolate	153 ± 43 nmol/L	3.8 ± 0.2	[66]
		Drink with 5.3 mg cocoa flavanols/kg body weight (young)	75 ± 7 nmol/L	1.4 ± 0.1	Drink with 5.3 mg cocoa flavanols/kg body weight (elderly)	72 ± 7 nmol/L	1.4 ± 0.1	[82]
		Drink with 10.7 mg cocoa flavanols/kg body weight (young)	176 ± 14 nmol/L	1.6 ± 0.1	Drink with 10.7 mg cocoa flavanols/kg body weight (elderly)	128 ± 11 nmol/L	1.8 ± 0.1	[82]
	4´-methyl-(-)-epicatechin	Low-flavanol cocoa drink	N.R.	N.R.	High-flavanol cocoa drink	N.R.	N.R.	[71]
	4´-*O*-methyl-(-)-epicatechin	Low-flavanol cocoa drink	25 ± 5 nmol/L	2	High-flavanol cocoa drink	41 ± 10 nmol/L	2	[61]
	4´-*O*-methyl-(-)-epicatechin 5-sulfate	N.R.	N.R.	N.R.	Dark chocolate	18 ± 6 nmol/L	3.8 ± 0.2	[66]
	4´-*O*-methyl-(-)-epicatechin 7-sulfate	N.R.	N.R.	N.R.	Dark chocolate	13 ± 4 nmol/L	3.8 ± 0.2	[66]
	4´-*O*-methyl-(-)-epicatechin-*O*-β-D-glucuronide	Low-flavanol cocoa drink	N.R.	N.R.	High-flavanol cocoa drink	N.R.	N.R.	[71]
		Low-flavanol cocoa drink	151 ± 46 nmol/L	2	High-flavanol cocoa drink	287 ± 58 nmol/L	2	[61]
	Epicatechin-*O*-β-D-glucuronide	Low-flavanol cocoa drink	N.R.	N.R.	High-flavanol cocoa drink	N.R.	N.R.	[71]
	Epicatechin-7-β-D-glucuronide	Low-flavanol cocoa drink	9 ± 3 nmol/L	2	High-flavanol cocoa drink	39 ± 13 nmol/L	2	[61]
	Catechin	Chocolate drink milk-free	0.21 ± 0.2 µmol/L	1–1.5	Chocolate drink with milk	0.20 ± 0.02 µmol/L	0.5–1	[74]
		No cocoa extract	0.00 µmol/L	0	Cocoa extract in water	0.16 ± 0.03 µmol/L	0.5–2	[68]
		N.R.	N.R.	N.R.	Cocoa based dairy drink	149 ± 18 nmol/L	2	[79]
		Low-flavanol cocoa drink	N.R.	N.R.	High-flavanol cocoa drink	N.R.	N.R.	[71]
		Low-flavanol cocoa drink	9 ± 3 nmol/L	2	High-flavanol cocoa drink	18 ± 3 nmol/L	2	[61]
	Epicatechin	N.R.	N.R.	N.R.	Cocoa based dairy drink	889 ± 114 nmol/L	2	[79]
		Chocolate drink milk-free	12.89 ± 0.95 µmol/L	1–2	Chocolate drink with milk	12.42± 0.97 µmol/L	0.5–1	[74]
		No cocoa extract	0.08 ± 0.46 µmol/L	0	Cocoa extract in water	5.96 ± 0.60 µmol/L	0.5–2	[68]
		Bread	19 ± 14 nmol/L	2	80 g of chocolate and 47 g of bread	355 ± 49 nmol/L	2	[63]
		Chocolate	0.15 ± 0.04 µmol/L	1–2	Cocoa	0.22 ± 0.06 µmol/L	0–1	[64]
Flavonoids and conjugates	Epicatechin	40 g of chocolate	103 ± 29 ng/mL	2.00 ± 0.00	80 g of chocolate	196 ± 57 ng/mL	2.57 ± 0.50	[62]
		Milk	N.D.	2	Milk and cocoa powder	625.7 ± 198.3 nmol/L	2	[70]
		Low-flavanol cocoa drink	N.R.	N.R.	High-flavanol cocoa drink	N.R.	N.R.	[71]
		Low-flavanol cocoa drink	3 ± 0 nmol/L	2	High-flavanol cocoa drink	19 ± 6 nmol/L	2	[61]
		Low-cocoa group	578 ± 61 nmol/L	2	High-cocoa group	N.R.	N.R.	[58]
	Epicatechin glucuronide	Chocolate	0.78 ± 0.28 µmol/L	1–2	Cocoa	0.91 ± 0.21 µmol/L	1–2	[64]
	Epicatechin sulfate	Chocolate	1.11 ± 0.43 µmol/L	0–1	Cocoa	1.14 ± 0.21 µmol/L	1–2	[64]
	Epicatechin sulfoglucuronide	Chocolate	1.19 ± 0.44 µmol/L	0–1	Cocoa	1.28 ± 0.76 µmol/L	0–1	[64]
	Methylated epicatechin sulfate	Chocolate	0.95 ± 0.27 µmol/L	1–2	Cocoa	1.00 ± 0.34 µmol/L	1–2	[64]
	Methylated epicatechin sulfoglucuronide	Chocolate	0.71 ± 0.14 µmol/L	1–2	Cocoa	0.78 ± 0.51 µmol/L	4–8	[64]
	*O*-methyl-(epi)-catechin-*O*-sulfate	Cocoa powder with water	60 ± 8 nmol/L	1.0 ± 0.2	Cocoa powder with milk	50 ± 8 nmol/L	1.3 ± 0.2	[77]
	Procyanidin B2	No cocoa extract	Detected	0	Chocolate extract in water	41 ± 4 nmol/L	0.5–2	[68]
		N.R.	N.R.	N.R.	Cocoa dairy drink	4.0 ± 0.6 nmol/L	2	[80]
Methylxanthines	3-methylxanthine	Cocoa powder with milk	0.6 ± 1.4 µmol/L	3.4 ± 3.3	Cocoa powder enriched with methylxanthines with milk	1.8 ± 0.9 µmol/L	2.2 ± 2.5	[81]
	7-methylxanthine	Cocoa powder with milk	2.1 ± 1.4 µmol/L	3.3 ± 1.6	Cocoa powder enriched with methylxanthines with milk	4.6 ± 1.8 µmol/L	4.5 ± 1.5	[81]
	Caffeine	Cocoa powder with milk	2.1 ± 1.3 µmol/L	2.1 ± 2.0	Cocoa powder enriched with methylxanthines with milk	12.1 ± 2.6 µmol/L	2.0 ± 1.1	[81]
		No chocolate dose	<2.5–21.4 µmol/L	0	Dark chocolate	4.6–25.3 µmol/L	1.5	[59]
	Paraxanthine	Cocoa powder with milk	9.5 ± 1.3 µmol/L	4.5 ± 2.9	Cocoa powder enriched with methylxanthines with milk	12.0 ± 2.2 µmol/L	3.5 ± 2.5	[81]
	Theobromine	No chocolate dose	<2.5–7.1 µmol/L	0	Dark chocolate	43.2–67.5 µmol/L	1.5	[59]
		Cocoa powder with milk	15.8 ± 3.3 µmol/L	1.9 ± 1.0	Cocoa powder enriched with methylxanthines with milk	51.9 ± 13 µmol/L	2.2 ± 1.1	[81]
Methylxanthines	Theobromine	Cocoa depleted control	N.R.	N.R.	Dark chocolate	70 µmol/L	3	[49]
		40 g of chocolate	6.365 ± 0.894 µg/mL	2.25 ± 0.88	80 g of chocolate	11.414 ± 1.190 µg/mL	3.25 ± 0.45	[62]
		Non-cocoa consumers	0.04–0.17 mg/kg	0–24	High cocoa consumers	0.33–0.66 mg/kg	0–24	[56]
		Baseline	2.3 ± 2.4 mg/L	0–12	Milk chocolate	7.6 ± 5.4 mg/L	0–12	[67]
		Baseline	2.3 ± 2.4 mg/L	0–12	Cocoa powder	19.3 ± 5.9 mg/L	0–12	[67]
		Baseline	2.3 ± 2.4 mg/L	0–12	Dark chocolate	30.6 ± 10.3 mg/L	0–12	[67]
	Theophylline	Cocoa powder with milk	11.5 ± 2.6 µmol/L	3.1 ± 3.2	Cocoa powder enriched with methylxanthines with milk	12.3 ± 4.3 µmol/L	1.9 ± 2.3	[81]
Phenolic acids	Ferulic acid	N.R.	N.R.	N.R.	Cocoa polymeric procyanidin concentrate	15 ± 2 ng/mL	3 ± 1	[60]

**Notes:** N.R. means non reported or non-cocoa matrix was used.

**Table 5:** Metabolites detected in plasma samples, that discriminated the baseline from treatments (enriched dark chocolate, dark chocolate and white chocolate), and times after intake where increase in measured signal was the highest, in an untargeted study [84].

Metabolite	Sampling Period (h)
β-hydroxybutyrate	6
Acetone	6
Acetoacetate	6
Aspartate	6
Lactate	2

**Table 6:** Metabolites analyzed in urine after acute cocoa interventions and studied with targeted methodologies.

Chemical Category	Metabolite	Treatment 1			Treatment 2			Reference
		Type of Matrix	Concentration	Sampling Period (h)	Type of Matrix	Concentration	Sampling Period (h)	
Flavonoids and conjugates	(-)-catechin	N.R.	N.R.	N.R.	Dairy cocoa drink	4.0 ± 0.4 µmol	0–24	[79]
	(-)-epicatechin	N.R.	N.R.	N.R.	Dairy cocoa drink	13 ± 2 µmol	0–24	[79]
		N.R.	N.R.	N.R.	Non dairy cocoa drink	N.R.	0–24	[76]
		N.R.	N.R.	N.R.	Cocoa polymeric procyanidin concentrate	0.6 ± 0.2 ng/mg creatintine	0–4	[60]
	(-)-epicatechin glucuronide	Non-dairy cocoa drink	194.95 µg/g creatinine	0–6	Dairy cocoa drink	112.79 µg/g creatinine	0–6	[75]
	(-)-epicatechin sulfate (isomer 1)	Non-dairy cocoa drink	48.83 µg/g creatinine	0–6	Dairy cocoa drink	30.86 µg/g creatinine	0–6	[72,75]
	(-)-epicatechin sulfate (isomer 2)	No- dairy cocoa drink	195.29 µg/g creatinine	6–12	Dairy cocoa drink	128.47 µg/g creatinine	6–12	[72,75]
	(-)-epicatechin sulfate (isomer 3)	Non-dairy cocoa drink	5.07 µg/g creatinine	0–6	Dairy cocoa drink	72.45 µg/g creatinine	0–6	[72,75]
	(-)-epicatechin-3´-sulfate	N.R.	N.R.	N.R.	Dark chocolate	5.80 ± 1.78 µmol	5–10	[66]
	(-)-epicatechin-3´-β-D-glucuronide	N.R.	N.R.	N.R.	Dark chocolate	8.74 ± 2.92 µmol	5–10	[66]
	(-)-epicatechin-4´-sulfate	N.R.	N.R.	N.R.	Dark chocolate	0.37 ± 0.13 µmol	5–10	[66]
	(-)-epicatechin-4´-β-D-glucuronide	N.R.	N.R.	N.R.	Dark chocolate	0.56 ± 0.14 µmol	5–10	[66]
	(-)-epicatechin-5-sulfate	N.R.	N.R.	N.R.	Dark chocolate	0.72 ± 0.36 µmol	5–10	[66]
	(-)-epicatechin-7-β-D-glucuronide	N.R.	N.R.	N.R.	Dark chocolate	4.59 ± 0.74 µmol	10–24	[66]
Flavonoids and conjugates	(-)-epicatechin-*O*-glucuronide	Cocoa powder with water	405 ± 44 µmol/L	0–2	Cocoa powder with milk	136 ± 24 µmol/L	2–5	[77]
	(-)-epicatechin-*O*-sulfate (isomer 2)	Cocoa powder with water	1127 ± 196 µmol/L	0–2	Cocoa powder with milk	737 ± 118 µmol/L	2–5	[77]
	(+)-catechin	N.R.	N.R.	N.R.	Dairy cocoa drink	9.8 ± 1.4 µmol	0–24	[79]
	(+)-epicatechin	N.R.	N.R.	N.R.	Dairy cocoa drink	10 ± 1 µmol	0–24	[79]
	Epicatechin	Hot water	N.D.	0–24	Cocoa powder with water	4.3 ± 4.2 µmol	0–24	[69]
	(epi) catechin-*O*-sulfate (isomer 1)	Cocoa powder with water	928 ± 110 µmol/L	0–2	Cocoa powder with milk	476 ± 75 µmol/L	0–2	[77]
	3´-*O*-methyl-(-)-epicatechin	N.R.	N.R.	N.R.	Cocoa polymeric procyanidin concentrate	0.2 ± 0.3 ng/mg creatinine	0–4	[60]
	3´-*O*-methyl-(-)-epicatechin 4´-sulfate	N.R.	N.R.	N.R.	Dark chocolate	1.27 ± 0.39 µmol	5–10	[66]
	3´-*O*-methyl-(-)-epicatechin 5-sulfate	N.R.	N.R.	N.R.	Dark chocolate	8.87 ± 3.05 µmol	5–10	[66]
	3´-*O*-methyl-(-)-epicatechin 7-sulfate	N.R.	N.R.	N.R.	Dark chocolate	1.55 ± 0.55 µmol	5–10	[66]
	3´-*O*-methyl-(-)-epicatechin-3´-β-D-glucuronide	N.R.	N.R.	N.R.	Dark chocolate	0.69 ± 0.12 µmol	5–10	[66]
	4´-*O*-methyl-(-)-epicatechin 5-sulfate	N.R.	N.R.	N.R.	Dark chocolate	0.92 ± 0.34 µmol	5–10	[66]
	4´-*O*-methyl-(-)-epicatechin 7-sulfate	N.R.	N.R.	N.R.	Dark chocolate	0.55 ± 0.29 µmol	5–10	[66]
	*O*-methyl-(epi)-catechin-*O-s*ulfate	Cocoa powder with water	1146 ± 231 µmol/L	0–2	Cocoa powder with milk	823 ± 131 µmol/L	0–2	[77]
Flavonoids and conjugates	Naringenin	Hot water	0.1 ± 0.2 µmol	0–24	Cocoa powder with water	0.3 ± 0.4 µmol	0–24	[69]
	Procyanidin B2	N.R.	N.R.	N.R.	Non-dairy cocoa drink	N.R.	0–24	[76]
Lignans	Enterodiol	N.R.	N.R.	N.R.	Non-dairy cocoa drink	N.R.	0–24	[76]
		Hot water	0.5 ± 1.1 µmol	0–24	Cocoa powder with water	0.2 ± 0.3 µmol	0–24	[69]
	Enterolactone	N.R.	N.R.	N.R.	Non-dairy cocoa drink	N.R.	0–24	[76]
		Hot water	3.8 ± 2.8 µmol	0–24	Cocoa powder with water	3.6 ± 2.9 µmol	0–24	[69]
Methylxanthines	1,3,7-trimethyluric acid	Cocoa powder with milk	0.7 ± 0.3 µmol/L	0–2	Cocoa powder with milk, enriched with methylxanthines	1.8 ± 0.6 µmol/L	9.7 ± 6.9	[81]
	1,3-dimethyluric acid	Cocoa powder with milk	1.3 ± 1.1 µmol/L	21.5 ± 6.0	Cocoa powder with milk, enriched with methylxanthines	3.4 ± 1.9 µmol/L	18.0 ± 7.5	[81]
	1,7-dimethyluric acid	Cocoa powder with milk	3.9 ± 2.7 µmol/L	20.0 ± 7.7	Cocoa powder with milk, enriched with methylxanthines	13.5 ± 7.8 µmol/L	12.7 ± 8.7	[81]
	1-methyluric acid	Cocoa powder with milk	9.2 ± 4.1 µmol/L	19.7 ± 8.2	Cocoa powder with milk, enriched with methylxanthines	45.2 ± 16.4 µmol/L	12.4 ± 7.5	[81]
	1-methylxanthine	Cocoa powder with milk	5.5 ± 2.7 µmol/L	21.2 ± 6.8	Cocoa powder with milk, enriched with methylxanthines	15.3 ± 7.3 µmol/L	13.7 ± 7.7	[81]
Methylxanthines	3,7-dimethyluric acid	Cocoa powder with milk	2.7 ± 1.1 µmol/L	21.2 ± 6.8	Cocoa powder with milk, enriched with methylxanthines	7.3 ± 2.9 µmol/L	14.3 ± 8.6	[81]
	3-methylxanthine	Cocoa powder with milk	34.8 ± 8.9 µmol/L	14.8 ± 9.1	Cocoa powder with milk, enriched with methylxanthines	98.1 ± 21.9 µmol/L	12.0 ± 7.4	[81]
	7-methylxanthine	Cocoa powder with milk	110.1 ± 40.1 µmol/L	16.3 ± 8.7	Cocoa powder with milk, enriched with methylxanthines	187.4 ± 82.1 µmol/L	12.0 ± 7.2	[81]
	Caffeine	Cocoa powder with milk	2.1 ± 0.7 µmol/L	14.5 ± 9.3	Cocoa powder with milk, enriched with methylxanthines	9.9 ± 3.5 µmol/L	7.7 ± 5.5	[81]
		Baseline	<2.5–23.4 µmol/L	0	Dark chocolate	4.2–24.7 µmol/L	1.5	[59]
	Paraxanthine	Cocoa powder with milk	3.5 ± 1.8 µmol/L	15.1 ± 9.0	Cocoa powder with milk, enriched with methylxanthines	10.7 ± 5.8 µmol/L	11.7 ± 7.5	[81]
	Theobromine	Cocoa powder with milk	50.4 ± 18.4 µmol/L	14.2 ± 9.6	Cocoa powder with milk, enriched with methylxanthines	177.4 ± 45.0 µmol/L	7.7 ± 5.5	[81]
		Baseline	2.5–94.9 µmol/L	0	Dark chocolate	131.9–449.4 µmol/L	1.5	[59]
	Theophylline	Baseline	1.0 ± 1.4 µmol/L	14.9 ± 8.9	Dark chocolate	2.5 ± 1.3 µmol/L	7.6 ± 5.8	[81]
*N*-phenylpropenoyl-L-amino acids	*N*-[3´,4´-dihydroxy-(E)-cinnamoyl]-L-aspartic acid	Baseline	N.D.	0	Non-dairy cocoa drink	4.76 ± 4.01 µg	2	[20]
	*N*-[3´,4´-dihydroxy-(E)-cinnamoyl]-L-dopa	Baseline	N.D.	0	Non-dairy cocoa drink	1.25 ± 1.19 µg	2	[20]
	*N*-[3´,4´-dihydroxy-(E)-cinnamoyl]-L-glutamic acid	Baseline	N.D.	0	Non-dairy cocoa drink	N.D.	N.D.	[20]
	*N*-[3´,4´-dihydroxy-(E)-cinnamoyl]-L-tyrosine	Baseline	N.D.	0	Non-dairy cocoa drink	0.30 ± 0.39 µg	2	[20]
	*N*-[4´-hydroxy-(E)-cinnamoyl]-L-aspartic acid	Baseline	N.D.	0	Non-dairy cocoa drink	64.37 ± 20.22 µg	2	[20]
	*N*-[4´-hydroxy-(E)-cinnamoyl]-L-dopa	Baseline	N.D.	0	Non-dairy cocoa drink	0.85 ± 0.96 µg	2	[20]
*N*-phenylpropenoyl-L-amino acids	*N*-[4´-hydroxy-(E)-cinnamoyl]-L-glutamic acid	Baseline	N.D.	0	Non-dairy cocoa drink	22.17 ± 12.57 µg	2	[20]
	*N*-[4´-hydroxy-(E)-cinnamoyl]-L-tryptophane	Baseline	N.D.	0	Non-dairy cocoa drink	<0.1 µg	1–4	[20]
	*N*-[4´-hydroxy-(E)-cinnamoyl]-L-tyrosine	Baseline	N.D.	0	Non-dairy cocoa drink	18.91 ± 5.53 µg	2	[20]
	*N*-[4´-hydroxy-3´-methoxy-(E)-cinnamoyl]-L-aspartic acid	Baseline	N.D.	0	Non-dairy cocoa drink	9.70 ± 2.93 µg	2	[20]
	*N*-[4´-hydroxy-3´-methoxy -(E)-cinnamoyl]-L-tyrosine	Baseline	N.D.	0	Non-dairy cocoa drink	1.91 ± 0.59 µg	2	[20]
	*N*-[cinnamoyl]-L-aspartic acid	Baseline	N.D.	0	Non-dairy cocoa drink	0.43 ± 0.33 µg	2	[20]
Phenolic acids and others	3-hydroxybenzoic acid	N.R.	N.R.	N.R.	Cocoa polymeric procyanidin concentrate	1 ± 1 ng/mg creatinine	0–4	[66]
		N.R.	N.R.	N.R.	Non-dairy cocoa drink	N.R.	0–24	[76]
	3,4-dihydroxyphenyl acetic acid	Baseline	15.8 ± 4.4 nmol/mg creatinine	−24–0	Chocolate	38.8 ± 12.3 nmol/mg creatinine	0–24	[65]
		Cocoa powder with water	1.60 ± 0.37 nmol/mg creatinine	6–12	Cocoa powder with milk	0.45 ± 0.10 nmol/mg creatinine	12–24	[78]
		N.R.	N.R.	N.R.	Cocoa polymeric procyanidin concentrate	10 ± 10 ng/mg creatinine	0–4	[60]
		N.R.	N.R.	N.R.	Non-dairy cocoa drink	N.R.	0–24	[76]
Phenolic acids and others	3,4-dihydroxyphenyl propionic acid	N.R.	N.R.	N.R.	Cocoa polymeric procyanidin concentrate	6 ± 5 ng/mg creatinine	0–4	[60]
		Baseline	3.1 ± 0.7 nmol/mg creatinine	−24–0	Chocolate	2.6 ± 0.6 nmol/mg creatinine	0–24	[65]
		Cocoa powder with water	16.84 ± 2.81 nmol/mg creatinine	0–6	Cocoa powder with milk	16.9 ± 4.0 nmol/mg creatinine	0–6	[78]
		N.R.	N.R.	N.R.	Non-dairy cocoa drink	N.R.	0–24	[76]
	3-hydroxyphenyl acetic acid	Cocoa powder with water	10.1 ± 3.42 nmol/mg creatinine	12–24	Cocoa powder with milk	7.36 ± 1.61 nmol/mg creatinine	12–24	[78]
		N.R.	N.R.	N.R.	Non-dairy cocoa drink	N.R.	0–24	[76]
		N.R.	N.R.	N.R.	Cocoa polymeric procyanidin concentrate	30 ± 20 ng/mg creatinine	0–4	[60]
	3-hydroxyphenyl propanoic acid	N.R.	N.R.	N.R.	Cocoa polymeric procyanidin concentrate	10 ± 7 ng/mg creatinine	0–4	[60]
	3-methoxy-4-hydroxy-phenylacetic acid	Cocoa powder with water	11.30 ± 1.63 nmol/mg creatinine	6–12	Cocoa powder with milk	9.30 ± 0.74 nmol/mg creatinine	6–12	[78]
		N.R.	N.R.	N.R.	Non-dairy cocoa drink	N.R.	0–24	[76]
	4-hydroxy-5-(3´,4´-dihydroxyphenyl) valeric acid	N.R.	N.R.	N.R.	Cocoa polymeric procyanidin concentrate	0.2 ± 0.3 ng/mg creatinine	0–4	[60]
	4-hydroxybenzoic acid	N.R.	N.R.	N.R.	Cocoa polymeric procyanidin concentrate	16 ± 6 ng/mg creatinine	0–4	[60]
		N.R.	N.R.	N.R.	Non-dairy cocoa drink	N.R.	0–24	[76]
	4-hydroxyphenyl acetic acid	N.R.	N.R.	N.R.	Cocoa polymeric procyanidin concentrate	210 ± 50 ng/mg creatinine	0–4	[60]
Phenolic acids and others	4-hydroxyphenyl propanoic acid	N.R.	N.R.	N.R.	Cocoa polymeric procyanidin concentrate	0.02 ± 0.04 ng/mg creatinine	0–4	[60]
	4-*O*-methylgallic acid	N.R.	N.R.	N.R.	Non-dairy cocoa drink	N.R.	0–24	[76]
		Hot water	N.D.	0–24	Cocoa powder with water	0.6 ± 1.1 µmol	0–24	[69]
	5-(3´,4´-dihydroxyphenyl) valerolactone	N.R.	N.R.	N.R.	Cocoa polymeric procyanidin concentrate	2 ± 4 ng/mg creatinine	48	[60]
	Caffeic acid	N.R.	N.R.	N.R.	Cocoa polymeric procyanidin concentrate	1.2 ± 0.5 ng/mg creatinine	0–4	[60]
		N.R.	N.R.	N.R.	Non-dairy cocoa drink	N.R.	0–24	[76]
		Hot water	0.1 ± 0.2 µmol	0–24	Cocoa powder with water	0.4 ± 0.4 µmol	0–24	[69]
	Dihydroxyphenyl valeric acid	N.R.	N.R.	N.R.	Cocoa polymeric procyanidin concentrate	0.01 ng/mg creatintine	-	[60]
	Ferulic acid	N.R.	N.R.	N.R.	Cocoa polymeric procyanidin concentrate	21 ± 4 ng/mg creatinine	0–4	[60]
		Baseline	131 ± 59 nmol/mg creatinine	−24–0	Chocolate	321 ± 99 nmol/mg creatinine	24–48	[65]
		N.R.	N.R.	N.R.	Non-dairy cocoa drink	N.R.	0–24	[76]
	Hippuric acid	Cocoa powder with water	193.16 ± 27.89 nmol/mg creatinine	0–6	Cocoa powder with milk	122.82 ± 18.83 nmol/mg creatinine	12–24	[78]
		N.R.	N.R.	N.R.	Non-dairy cocoa drink	N.R.	0–24	[76]
		Baseline	2943 ± 1923 nmol/mg creatinine	−24–0	Chocolate	974± 115 nmol/mg creatinine	0–24	[65]
	*m*-coumaric acid	N.R.	N.R.	N.R.	Cocoa polymeric procyanidin concentrate	1 ± 1 ng/mg creatinine	0–4	[60]
		Hot water	0.5 ± 0.9 µmol	0–24	Cocoa powder with water	1.4 ± 1.4 µmol	0–24	[69]
Phenolic acids and others	*m*-coumaric acid	N.R.	N.R.	N.R.	Non-dairy cocoa drink	N.R.	0–24	[76]
	Methyl-5-(3´, 4´-dihydroxyphenyl)-valerolactone	N.R.	N.R.	N.R.	Cocoa polymeric procyanidin concentrate	0.5 ± 0.8 ng/mg creatinine	48	[60]
	*m*-hydroxybenzoic acid	Baseline	Non detected	−24–0	Chocolate	8.93 ± 3.9 nmol/mg creatinine	24–48	[65]
		Cocoa powder with water	0.56 ± 24 nmol/mg creatinine	6–12	Cocoa powder with milk	0.60 ± 0.28 nmol/mg creatinine	12–24	[78]
	*m*-hydroxyphenyl acetic acid	Baseline	21.2 ± 3.5 nmol/mg creatinine	−24–0	Chocolate	156 ± 54 nmol/mg creatinine	24–48	[65]
	*m*-hydroxyphenyl propionic acid	Baseline	5.4 ± 2.4 nmol/mg creatinine	−24–0	Chocolate	13.4 ± 4.1 nmol/mg creatinine	24–48	[65]
	*p*-coumaric acid	N.R.	N.R.	N.R.	Cocoa procyanidin concentrate	1 ± 1 ng/mg creatinine	48	[60]
		N.R.	N.R.	N.R.	Non-dairy cocoa drink	N.R.	0–24	[76]
	Phenylacetic acid	Cocoa powder with water	163.77 ± 22.10 nmol/mg creatinine	0–6	Cocoa powder with milk	167.72 ± 23.80 nmol/mg creatinine	0–6	[78]
		Baseline	70.1 ± 11.3 nmol/mg creatinine	−24–0	Chocolate	36.8 ± 8.1 nmol/mg creatinine	24–48	[65]
	*p*-hydroxybenzoic acid	Baseline	54.2 ± 8.1 nmol/mg creatinine	−24–0	Chocolate	41.2 ± 10.4 nmol/mg creatinine	24–48	[65]
		Cocoa powder with water	6.39 ± 0.66 nmol/mg creatinine	0–6	Cocoa powder with milk	3.01 ± 0.97 nmol/mg creatinine	12–24	[78]
Phenolic acids and others	*p*-hydroxyhippuric acid	Cocoa powder with water	3.13 ± 0.60 nmol/mg creatinine	0–6	Cocoa powder with milk	1.76 ± 0.30 nmol/mg creatinine	6–12	[78]
		Baseline	71.1 ± 13.6 nmol/mg creatinine	−24–0	Chocolate	90.5 ± 21.3 nmol/mg creatinine	24–48	[65]
		N.R.	N.R.	N.R.	Non-dairy cocoa drink	N.R.	0–24	[76]
	Protocatechuic acid	Cocoa powder with water	11.07 ± 1.19 nmol/mg creatinine	0–6	Cocoa powder with milk	8.8 ± 2.2 nmol/mg creatinine	0–6	[78]
		N.R.	N.R.	N.R.	Non-dairy cocoa drink	N.R.	0–24	[76]
		N.R.	N.R.	N.R.	Cocoa polymeric procyanidin concentrate	40 ± 20 ng/mg creatinine	8–24	[60]
	Vanillic acid	Cocoa powder with water	5.96 ± 1.15 nmol/mg creatinine	0–6	Cocoa powder with milk	9.85 ± 1.27 nmol/mg creatinine	0–6	[78]
		Baseline	64.6 ± 25.5 nmol/mg creatinine	−24–0	Chocolate	228 ± 33 nmol/mg creatinine	0–24	[65]
		N.R.	N.R.	N.R.	Non-dairy cocoa drink	N.R.	0–24	[76]
		N.R.	N.R.	N.R.	Cocoa polymeric procyanidin concentrate	14 ± 4 ng/mg creatinine	0–4	[60]

**Notes:** N.R. refers to non-reported or non-cocoa matrix was used, and N.D. to non-detected.

**Table 7:** Metabolites analyzed in urine after acute cocoa intervention studied with non-targeted methodologies.

Period (h)	Purine Metabolites	Polyphenol Metabolites	Nicotinic Acid Metabolites	Amino Acid Metabolites	Others	Reference
Baseline	*N*-methylguanine	-	-	-	-	[87]
0–6	3-methyluric acid 3-methylxanthine 3,7-dimethyluric acid 7-methyluric acid 7-methylxanthineAMMU (4) Caffeine Theobromine	3´-methoxy-4´-hydroxyphenyl valerolactone 3´-methoxy-4´-hydroxyphenyl valerolactone glucuronide 4-hydroxy-5-(3,4-dihydroxyphenyl)-valeric acid 5-(3´,4´-dihydroxyphenyl)-γ-valerolactone glucuronide Epicatechin-*O—*sulfate *O*-methyl epicatechin Vanillic acid Vanilloylglycine	Hydroxynicotinic acid Trigonelline	Tyrosine	3,5-diethyl-2-methyl pyrazine Cyclo(Ser-Tyr) Cyclo(Pro-Pro) Hydroxyacetophenone	[86]
	3-methylxanthine 7-methylxanthine Theobromine	Vanilloylglycine	-	-	-	[87]
	3-methyluric acid 3-methylxanthine 3,7- dimethyluric acid 7-methyluric acid 7-methylxanthine AMMU Caffeine Theobromine	3´-methoxy-4´-hydroxyphenyl valerolactone 3´-methoxy-4´-hydroxyphenyl valerolactone glucuronide 4-hydroxy-5-(3,4-dihydroxyphenyl)-valeric acid 5-(3´,4´-dihydroxyphenyl)--valerolactone sulfate 5-(3´,4´-dihydroxyphenyl)-valerolactone glucuronide Hippurate Epicatechin-*O*-sulfate Vanilloylglycine	Hydroxynicotinic acid *N*1-methylnicotinamide	Alanine Arginine Glycine Tyrosine Valine	2-hydroxyisobutyrate 3-hydroxyisobutyrate 3-hydroxyisovalerate 4-hydroxyphenyl acetate Creatinine Dimethylamine Lactate *O*-feruoylquinate Pyruvate	[84]
6–12	3-methyluric acid 3-methylxanthine 3,7-dimethyluric acid 7-methyluric acid 7-methylxanthine AMMU Caffeine Theobromine	3´-methoxy-4´-hydroxyphenyl valerolactone 3´-methoxy-4´-hydroxyphenyl valerolactone glucuronide 4-hydroxy-5-(3,4-dihydroxyphenyl)-valeric acid 5-(3´,4´-dihydroxyphenyl)-g-valerolactone glucuronide 5-(3´,4´-dihydroxyphenyl)-γ-valerolactone sulfate 5-(3´,4´-dihydroxyphenyl)-γ-valerolactone glucuronide	-	-	Cyclo(Ser-Tyr) 3,5-diethyl-2-methyl pyrazine	[86]
	3-methylxanthine 7-methylxanthine Theobromine	Dihydroxyphenyl valerolactone glucuronide	-	-	Furoylglycine	[87]
12–24	3-methyluric acid 3-methylxanthine 3,7-dimethyluric acid 7-methyluric acid 7-methylxanthine AMMU Theobromine	3´-methoxy-4´-hydroxyphenyl valerolactone 3´-methoxy-4´-hydroxyphenyl valerolactone glucuronide 4-hydroxy-5-(3,4-dihydroxyphenyl)-valeric acid 5-(3´,4´-dihydroxyphenyl)-g-valerolactone glucuronide 5-(3´,4´-dihydroxyphenyl)-γ-valerolactone glucuronide	-	-	Cyclo(Ser-Tyr)	[86]
	3-methylxanthine 7-methylxanthine Theobromine	-	-	Xanthurenic acid	-	[87]

**Notes:** (4) AMMU means 6-amino-5-[*N*-methylformylamino]-1-methyluracil.

**Table 8:** Metabolites analyzed in urine and plasma after acute, short-term, or regular cocoa intake interventions, studied with non-targeted and targeted methodologies.

Type of Metabolite	Metabolites in Urine		Metabolites in Plasma (Targeted)	
	Non-Targeted [55]	Targeted [57]	[57]	[58]
Polyphenols metabolites	(DHPV) 5-(3´,4´-dihydroxphenyl)-valerolactone sulfoglucuronide (HDHPVA) 4-hydroxy-5-(dihydroxyphenyl)-valeric acid glucuronide (HHMPVA) 4-hydroxy-5-(hydroxyl-methoxyphenyl)-valeric acid glucuronide (HHPVA) 4-hydroxy-5-(hydroxyphenyl)-valeric acid sulphate (HPV) hydroxyphenyl-valerolactone glucuronide (HPVA) 4-hydroxy-5-(phenyl)-valeric acidsulphate (MHPV) methoxyhydroxyphenyl valerolactone DHPV glucuronide (1) and (2) DHPV sulphate (epi) catechin glucuronide (epi) catechin sulphate HDHPVA HDHPVA sulphate HHPMVA sulphate HPV sulphate MHPV glucuronide Vanillic acid Vanillin sulphate	(-)-epicatechin 3-hydroxybenzoic acid 3-hydroxyhippuric acid 3-hydroxyphenyl acetic acid 3-hydroxyphenyl propionic acid 3-methoxy-4-hydroxyphenyl acetic acid 3,4-dihydroxyphenyl acetic acid 3,4-dihydroxyphenyl propionic acid4-hydroxybenzoic acid 4-hydroxyhippuric acid5-(3´,4´-dihydroxyphenyl)-γ-valerolactone 5-(3´-methoxy, 4´-hydroxyphenyl)-γ-valerolactone *O*-glucuronide 5-(3´-methoxy, 4´-hydroxyphenyl)-γ-valerolactone *O*-sulfate 5-(3´,4´-dihydroxyphenyl)-γ-valerolactone *O*-glucuronide isomers Caffeic acid Epicatechin-3´-*O*-glucuronide Epicatechin-7-*O*-glucuronide Epicatechin-*O*-glucuronide isomers Epicatechin-*O*-sulfate isomers Ferulic acid*m*-coumaric acid*O*-methyl-epicatechin-*O*-glucuronide isomers*O*-methyl-epicatechin-*O*-sulfate isomers*p*-coumaric acid Phenylacetic acid Protocatechuic acid Vanillic acid	3-hydroxyhippuric acid 3-hydroxyphenyl acetic acid 3-hydroxyphenyl propionic acid 3,4-dihydroxyphenyl acetic acid 3,4-dihydroxyphenyl propionic acid 4-hydroxybenzoic acid 4-hydroxyhippuric acid 5-(3´,4´-dihydroxyphenyl)-γ-valerolactone 5-(3´-methoxy, 4´-hydroxyphenyl)-γ-valerolactone *O*-glucuronide 5-(3´,4´-dihydroxyphenyl)-γ-valerolactone *O*-glucuronide (1) and (2) Caffeic acid Ferulic acid *p*-coumaric acid Phenylacetic acid Protocatechuic acid Vanillic acid	3´-*O*-methyl-(-)-epicatechin 4´-*O*-methyl-(-)-epicatechin Epicatechin
Purine metabolites	3-methyluric acid 3,7-dimethyluric acid 7-methylxanthine AMMU 1 and 2 (5) Theobromine Xanthine	-	-	-
Others	Aspartyl-phenylalanine Cyclo(aspartyl-phenylalanyl) Furoylglycine Methylglutaryl carnitine	-	-	-

**Notes:** (5) 1 and 2 correspond to isomers AMMU= 6-amino-5-[*N*-methylformylamino]-1-methyluracil.
